# Effect of Process Parameters on Flow Length and Flash Formation in Injection Moulding of High Aspect Ratio Polymeric Micro Features

**DOI:** 10.3390/mi9020058

**Published:** 2018-01-31

**Authors:** Abdelkhalik Eladl, Rania Mostafa, Aminul Islam, Dario Loaldi, Hassan Soltan, Hans N. Hansen, Guido Tosello

**Affiliations:** 1 Department of Mechanical Engineering, Technical University of Denmark, Produktionstorvet, DTU Building 427A, DK-2800 Kongens Lyngby, Denmark; abdelkhaliq@mans.edu.eg (A.E.); mais@mek.dtu.dk (A.I.); darloa@mek.dtu.dk (D.L.); hnha@mek.dtu.dk (H.N.H.); 2 Faculty of Engineering, Production Engineering and Mechanical Design Department, Mansoura University, Elgomhouria St., Mansoura City 35516, Egypt; ranmos75@mans.edu.eg (R.M.); hasoltan@mans.edu.eg (H.S.)

**Keywords:** micro injection moulding, design of experiments, part mass, flow length, flash formation

## Abstract

This paper reports an investigation of the effects of process parameters on the quality characteristics of polymeric parts produced by micro injection moulding (μIM) with two different materials. Four injection moulding process parameters (injection velocity, holding pressure, melt temperature and mould temperature) were investigated using Polypropylene (PP) and Acrylonitrile Butadiene Styrene (ABS). Three key characteristics of the mouldings were evaluated with respect to process settings and the material employed: part mass, flow length and flash formation. The experimentation employs a test part with four micro fingers with different aspect ratios (from 21 up to 150) and was carried out according to the Design of Experiments (DOE) statistical technique. The results show that holding pressure and injection velocity are the most influential parameters on part mass with a direct effect for both materials. Both parameters have a similar effect on flow length for both PP and ABS at all aspect ratios and have higher effects as the feature thickness decreased below 300 μm. The study shows that for the investigated materials the injection speed and packing pressure were the most influential parameters for increasing the amount of flash formation, with relative effects consistent for both materials. Higher melt and mould temperatures settings were less influential parameters for increasing the flash amount when moulding with both materials. Of the two investigated materials, PP was the one exhibiting more flash formation as compared with ABS, when corresponding injection moulding parameters settings for both materials were considered.

## 1. Introduction

For the mass production of micro products, micro injection moulding (μIM) represents one of the most important manufacturing processes because it matches the capabilities of a low-cost process and the requirements of micro products, such as dimensions in the sub-millimetre range and low tolerances (in the order of few micrometres down to the sub-micrometre range). Components manufactured successfully by micro injection moulding find applications into the following main sectors: medical and biomedical, automotive industry, telecommunication area, IT components and aerospace. In all these applications, the replication of component micro features is a key issue which determines the reliability of the selected manufacturing route. Replication fidelity depends greatly on the feature size, aspect ratio and surface area.

Quality characteristics in μIM are usually associated with the ability to completely fill the micro scale features in the mould cavity during processing. To improve the quality of injection moulded micro components many research groups worldwide have investigated in recent years different factors affecting the replication capabilities of the process. In particular, this includes research in process optimization, material rheology, design and manufacture of tools and development of new tooling technologies [1].

Micro cantilevers are one of the key elements in micro sensors (e.g., biochemical sensors, calorimetry sensors, humidity sensors, accelerometers, atomic force microscopes, etc.) because they are sensitive to small changes in mass and temperature. Polymeric micro cantilever structures with high aspect ratios (i.e., the ratios between the length and thickness of a cantilever finger), can be fabricated by filling with polymer melt the micro cantilever/fingers cavities. While the cross-sectional area decreases, the flow length becomes increasingly limited because the melt cools and solidifies rapidly in the cavities. This is caused by the increased surface-to-volume ratio and is common for other micro scale parts. The filling process in micro injection moulding is more complex because of peculiar phenomena happening at the micro scale such as high heat transfer at the melt-mould wall interface, presence of wall slip, influence of surface tension, compressibility of the melt and pressure-dependent viscosity. All the aspects that have been reported are affecting μIM at levels that are different from those encountered in injection moulding of conventionally-sized components [2].

Many experimental and theoretical studies have been conducted to determine the most influential factors for improving filling performance in micro injection moulding. Most studies tried to find the relationship between the process parameters and the achievable filling length, or the replication quality. The main factors investigated by researchers have been: melt and mould temperature, injection velocity, hold pressure and holding time. Their direct effect on the melt flow property and flow status is proven in conventional injection moulding.

The importance of those factors has been assessed for different applications, including microfluidic systems [3,4], micro channel moulding optimization [5,6,7], replication of micro ribs and micro features [8,9,10,11,12,13,14,15,16,17,18,19,20] and the effects of the processing parameters on micro cantilever using micro fingers dimensions as output response [1,2,21,22].

In literature, the filling mechanism of micro injection moulding appeared to be still not fully understood. The main conclusions from these studies show little agreement among them. In particular, there is a limited accordance with respect to which parameters have the most influence on the quality of the part. Each study has found a different set of significant parameters. This is probably due to the fact that different experimental investigations have employed tools with different mould design features (i.e., sprue and runner dimensions, gate design, cooling/heating layout, etc.) as well as different cavity geometry design. Further causes for limited agreement lie on the fact that different materials have been used. Therefore, the process windows employed are not only set at different levels but have different extensions, i.e., the same parameters investigated in different studies are varied within different intervals. Last but not least, also data treatment may have an influence, particularly for multi-variate analysis and in presence of interactions among parameters. For examples, in some of the studies found in literature, e.g., [1,6,7,9,11,21,22], research is carried out either by using the DOE technique or by using the one-factor-at-the-time approach to study the effect of a set of process parameters on a response.

Nevertheless, a shared result among the recent research trends [3,11,17,19,21,22], indicates that one of the major factor influencing both process significance factors and the replication quality of micro injected components is the aspect ratio, intended as the height/length ratio, of the designed and replicated features.

The purpose of this study is to improve the understanding of the behaviour of polymer melt filling through micro cantilever/fingers cavities and to study the correlation between flow length and flash formation using a unified DOE approach for both an amorphous polymer (Acrylonitrile Butadiene Styrene) and a semi-crystalline polymer (Polypropylene). Both materials could be moulded using the same process window, therefore allowing for an understanding of replication and flash formation behaviour related to the actual material properties. The experiments examined the effects of four process parameters and four different aspect ratio 21, 30, 50, 150 in replicating micro fingers with both polymer materials using part mass, flow length and flash formation as part quality outputs in order to identify which process parameters are the most influential during the filling stage of the micro injection moulding process.

## 2. Materials and Methods

### 2.1. Polymeric Materials

**Figure 1:**
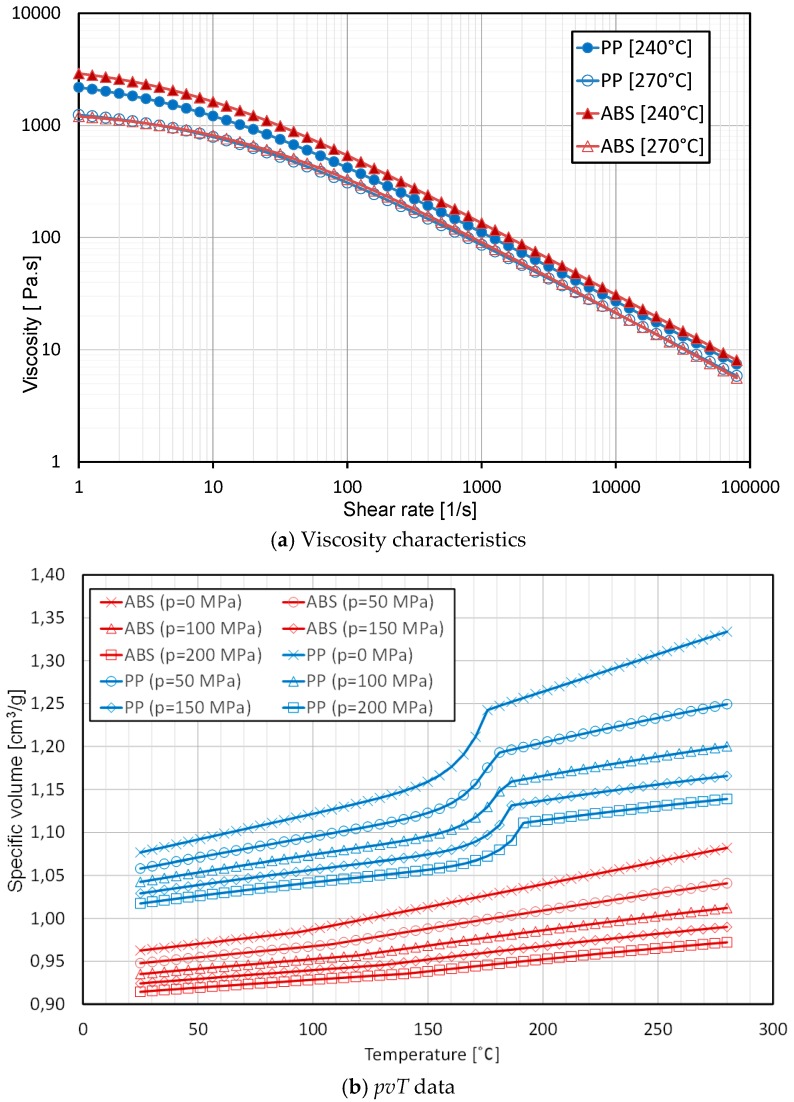
(**a**) viscosity characteristics and (**b**) *pvT* data of the two polymers [23].

Two commercially available unfilled materials used in conventional injection moulding (IM) such as Polypropylene (PP, trade name 400-GA05), manufactured by INEOS Olefins Polymers Europe (London, UK) and Acrylonitrile Butadiene Styrene (ABS, trade name Terluran GR35), manufactured by BASF (Ludwigshafen, Germany), were selected for the experiments. Figure 1 shows the viscosity characteristics and *pvT* data (pressure, specific volume and temperature) for the two polymers. Each polymer went through desiccant drying and dehumidifying cycles before the injection moulding trials, to remove any surface or absorbed moisture.

### 2.2. Part Geometry and Tooling

**Figure 2:**
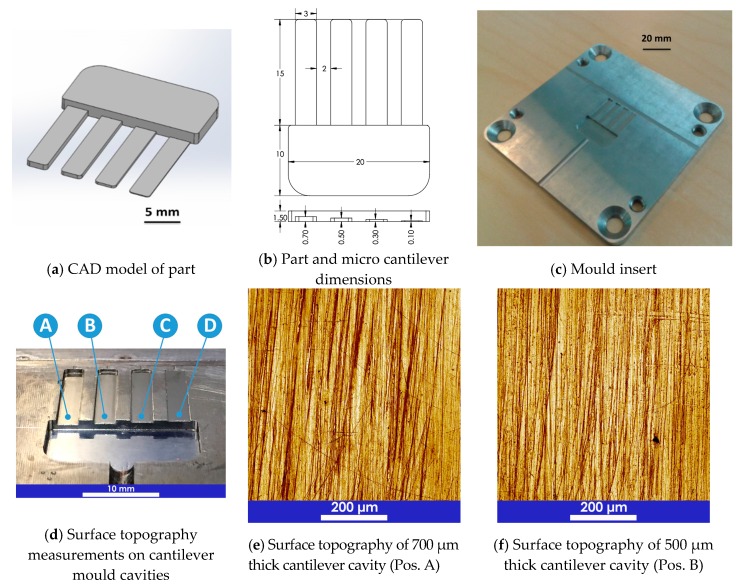

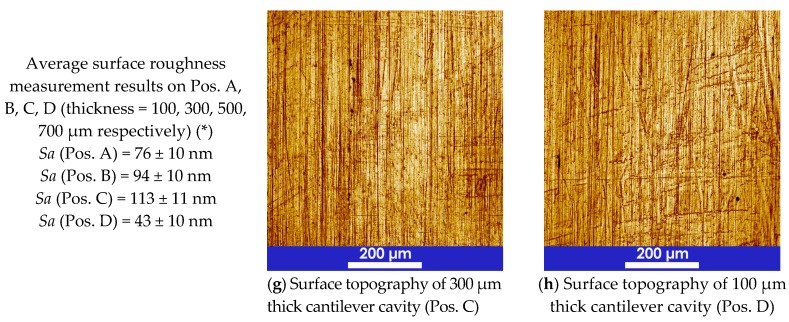
Model, dimensions, mould insert, cavity surface topographies and roughness of the micro finger test structure. Surface scanning area = 644 μm × 644 μm (4096 × 4096 pixels), acquisitions obtained with laser confocal microscope using a 20× magnification objective (numerical aperture = 0.60). (*) Interval indicates estimated measurement uncertainty including repeatability, resolution and instrument calibration.

The main body of the part had a rectangular shape (20 mm × 10 mm × 1.5 mm) with four cantilever/fingers. The four fingers had the same length, 15 mm, the same width, 3 mm and different thicknesses: 700 μm, 500 μm, 300 μm, 100 μm. Figure 2 illustrates all the dimensions of the micro-fingers and the test structure. The main body adjoining the four cantilever/fingers had a depth of 1.5 mm. This is where the melted polymer expanded after passing through the gate and homogenized before entering into the cantilevers. Four venting channels were placed at the end of each cantilever to improve air evacuation. The outside dimensions of the mould insert were 85 mm × 85 mm × 4 mm and the material was pre-hardened tool steel. A single open gate design was used. The gate had the same depth as the cross-sectional thickness of the main body, thus reducing the flow resistance and premature freeze-off of the gate. The test geometry was designed in such a way that it facilitated a relatively simple measurement procedure of the moulded cantilevers thus reducing any error source coming from the measuring strategy.

A high-speed precision milling process was used to generate the cavities. Afterwards, a polishing process was applied to the cavity walls in order to obtain a surface roughness in the range of 40–110 nm (see Figure 2). Such level of roughness ensured that the polymer flow was not affected by the presence of surface topography, even for very thin cavities as in the case of the cantilevers considered in this study. Recent studies on the effect of surface roughness on the polymer flow on micro cavities have shown that the mould surface topography affects the flow length when the average surface roughness in in the range of 500 nm to 10 μm for thicknesses of 50 μm, 100 μm, 200 μm, 250 μm, 1000 μm, corresponding to a roughness-to-thickness ratio of 0.25% up to 4.0% [24,25,26]. On the contrary, mould surface roughness in the range between 40 nm and 170 nm on cavities with thickness in the range between 250 μm to 1000 μm, corresponding to a roughness-to-thickness ratio of 0.01% up to 0.02% has shown to have no influence and no statistical significance on the polymer flow length [25,26].

### 2.3. Experimentation

Injection moulding experiments were carried out on an Arburg (Loßburg, Germany) Allrounder 370A injection moulding machine which has a maximum clamping force of 600 KN and a screw diameter of 18 mm. The investigated parameters were injection velocity (V_i_), holding pressure (P_h_), melt temperature (T_b_) and mould temperature (T_m_). These parameters were considered as factors affecting the optimization of the micro injection moulding process. The criteria used for selecting the maximum and minimum values of these process parameters took into account the equipment characteristics, process feasibility and materials characteristics. The process parameters levels were as follows:

- Melt temperature:
  - (Min) 240 °C was selected as the lowest melt temperature following the recommendation of the material supplier.
  - (Max) 270 °C was selected as the highest melt temperature following the recommendation of the material supplier.
- Mould temperature:
  - (Min) 20 °C was the lowest temperature allowing micro finger replication and complete filling of the cavity.
  - (Max) 60 °C was selected as the highest melt temperature following the recommendation of the material supplier, in order to allow successful demoulding of the part from the cavity.
- Holding pressure:
  - (Min) 10 MPa for PP materials and 20 MPa for ABS materials, which provided acceptable shrinkage compensation and dimensional accuracy.
  - (Max) 60 MPa for PP materials and 70 MPa for ABS materials were the maximum values allowing automatic demoulding without distortion.
- Injection velocity:
  - (Min) 140 mm/s was selected based on experimentation. This was the minimum injection speed that allowed the part to fill completely.
  - (Max) 220 mm/s was selected based on experimentation. This was the highest injection speed at which a continuous injection moulding process could be achieved.

The design of experiment (DOE) approach was applied to assess the effects of four parameters, each varying between a maximum and minimum value. This approach allowed all investigated process parameters to be taken into account simultaneously, in assessing their main effects. In this way, it was possible to systematically investigate process and/or product related variables that influenced the product and/or process quality, respectively. In particular, process conditions and part characteristics that affect product quality and cost could be identified, in order to improve the product manufacturability, quality, reliability and production quantities [27].

Factorial design is frequently used in experiments involving several factors and when it is necessary to study the factors main effects and interactions on various responses. Two different approaches can be distinguished when implementing DOE studies: full-factorial design, widely used when it is necessary to investigate the joint effects of several factors on a response and fractional factorial designs that are applied to reduce experimental efforts of large DOE studies, mostly for screening purposes. In this research, a two-level four-factor full-factorial design with resolution V (2^4^) was applied, scheduling 16 treatments, which were carried out as shown in Table 1. The 16 treatments were executed following a randomly ordered succession.

**Figure 3:**
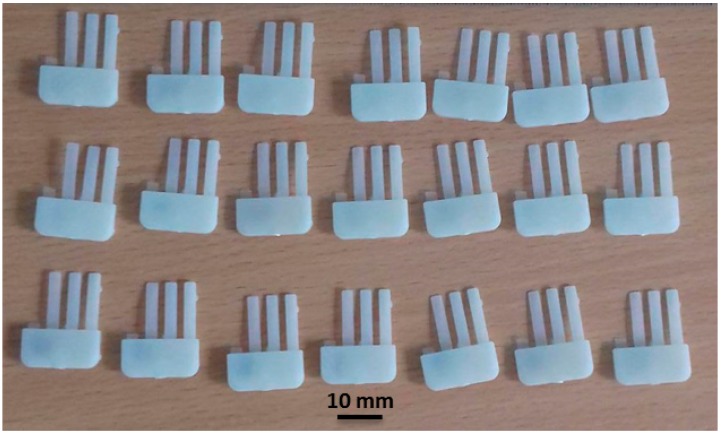
21 moulded samples from one DOE configuration.

For each run, the machine was run to firstly complete 50 continuous cycles in order to stabilize the process, then the following 21 parts were collected and numbered for subsequent inspection. Figure 3 shows a photograph of the 21 moulded samples from one of the DOE settings that were used for the analyses. Given that two different materials were considered, two DOE full-factorial designs with resolution V (2^4^) were applied for both materials. In addition, 21 trials were performed for each combination of controlled parameters. Thus, in total 21 × 16 × 2 = 672 experimental trials were carried out.

The mass of each moulded part and the runner mass were measured using a sensitive scale with a resolution of 0.01 mg and the averages of the measured masses were recorded and analysed. The flow lengths were measured for the different aspect ratio features using an optical quality control CNC coordinate measuring machine (CMM) having a resolution of 0.5 μm and an accuracy 4 μm.

## 3. Analysis of the Results

### 3.1. Factors Affecting Part Mass

**Figure 4:**
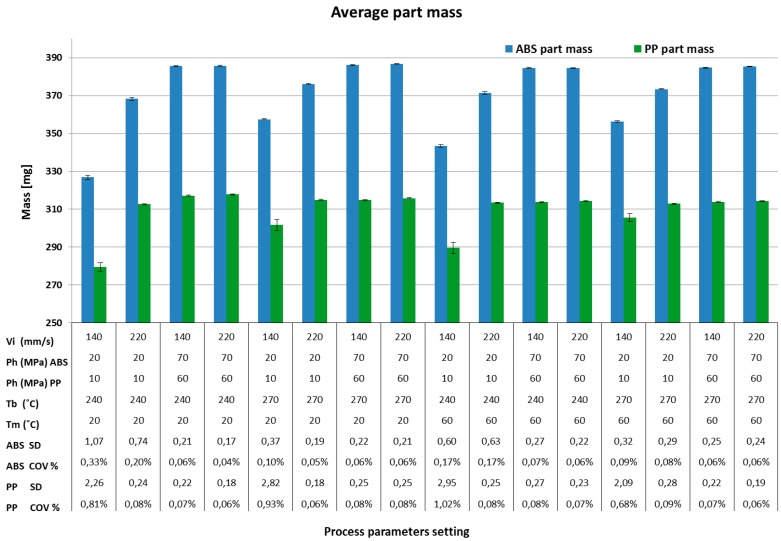
Average part mass for each experimental run for both materials ABS and PP. Note 1: error bars on histograms indicate standard deviation (SD). Note 2: Coefficient of Variation COV% = average/SD × 100%.

Complete mould filling and process stability are among the most important quality criteria for process quality assessment and are here represented by part mass. Figure 4 shows the effect of process parameters in the 16 DOE combinations on average part mass for both materials. It shows that the average mass of both materials had the same trends across the 16 treatments. Maximum mass for both materials was obtained at treatments 4, 8, 12, 16 which had a high level of processing parameters. On the other hand, minimum mass for both materials was obtained at 1, 5, 9 and 13, which had a low level of the processing parameters. ABS materials yielded a larger mass than PP materials for all the 16 treatments due to the difference in specific volume, as clearly indicated by the *pvT* plot represented in Figure 1b.

**Figure 5:**
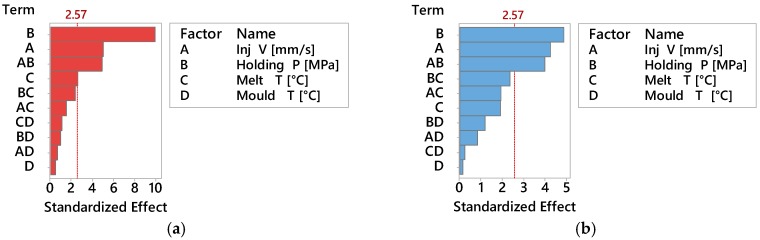
Pareto chart of the Standardized Effects for ABS (**a**) and PP (**b**), on average Part Mass (α = 0.05).

**Figure 6:**
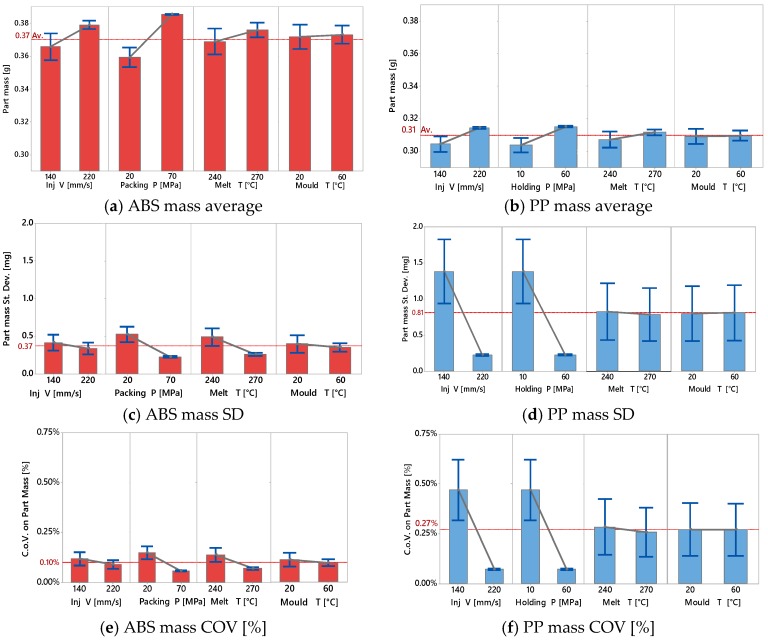
Main effects plot of: average part mass for ABS (**a**) and PP (**b**), standard deviation of part mass for ABS (**c**) and PP (**d**), coefficient of variation for ABS (**e**) and PP (**f**).

Figure 5 shows the Pareto charts of the standardized effect on the average mass for ABS (Figure 5a) and PP (Figure 5b) considering a significance of α = 0.05. The effect of holding pressure was the larger on the results for both materials. A remarkable effect was given also by injection velocity and its second order interaction with holding pressure, also common to both materials. In Figure 6 are also represented the main effect plots corresponding to part mass as responses for ABS and PP materials respectively. The effect of holding pressure on part mass was expected, since increasing the holding pressure allowed for more material to fill the mould cavity and to compensate for shrinkage before complete freezing, hence increasing its mass.

Increased injection velocity was shown to be a source of increasing part mass. This is due to the fact that higher velocity led to an increase in shear rate, which in turn decreased the viscosity of the polymer and allows for improved flow inside the mould cavity. Furthermore, higher injection velocity implied higher maximum injection pressure, which increased the quantity of material injected due to material compressibility (also visible in the *pvT* plot represented in Figure 1b).

For these reasons, the effect of injection velocity on the average mass was an increase of 3.6% and 3.3% for ABS and PP parts respectively.

Higher process settings resulted on higher repeatability with the same trends and dependency on the four parameters for both materials. A low level of injection velocity also reduced the process stability. In Figure 6c and especially Figure 6d the main effects plots of the measured mass samples show that the process standard deviation is reduced from 1.4 mg to 0.2 mg for PP and from 0.4 mg to 0.3 mg in the case of ABS. Optimal process conditions could lead to a relative standard deviation (i.e., coefficient of variation = average/standard deviation) of 0.1% or below, as shown in Figure 6e,f.

### 3.2. Factors Affecting Finger Filling Flow Length

**Figure 7:**
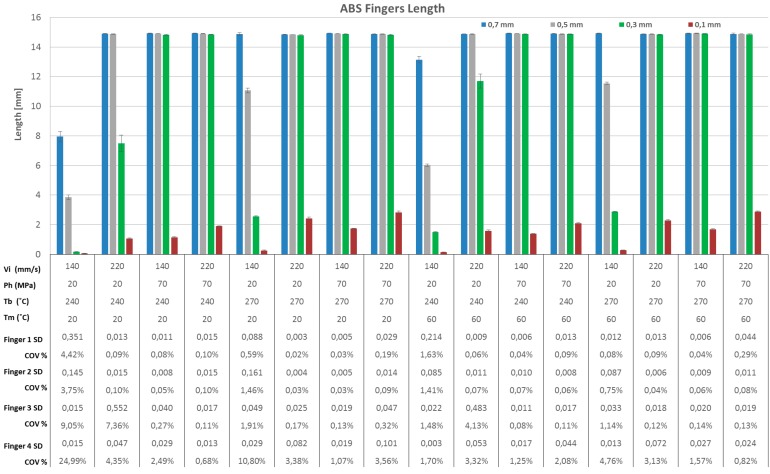
Average flow length of ABS on the four fingers for each DOE experimental run. Note 1: error bars on histograms indicate standard deviation (SD). Note 2: Coefficient of Variation COV% = average/SD × 100%.

**Figure 8:**
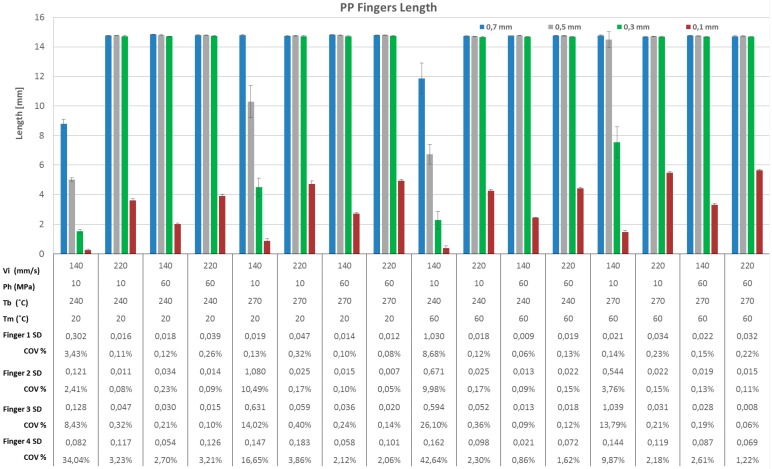
Average flow length of PP on the four fingers for each DOE experimental run. Note 1: error bars on histograms indicate standard deviation (SD). Note 2: Coefficient of Variation COV% = average/SD × 100%.

**Figure 9:**
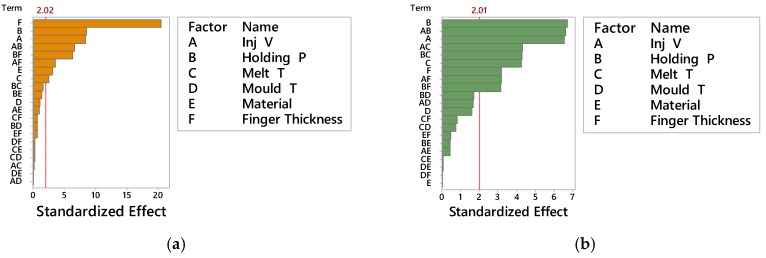
Pareto chart of the Standardized Effects for short Finger Thickness (100–300 μm) (**a**) and long Finger Thickness (500–700 μm) (**b**), on average Flow length (α = 0.05).

Flow length is one of the most important quality criteria in injection moulding and it is particularly critical with micro scale geometries with thin walls and high aspect ratios. Flow length was used in this research to evaluate the filling capacity of the moulding system, especially with respect to the aspect ratio (A/R), i.e., the ratio between the length of the finger and its thickness. The different aspect ratios studied were 21, 30, 50 and 150 respectively. Figure 7 and Figure 8 show a histogram chart of the four fingers average filled length in each experimental run for ABS and PP respectively. Figure 9 shows the main effect plots for filled lengths with respect to processing parameters and fingers thickness for both ABS and PP materials.

From Figure 7 and Figure 8 it can be seen that the length of finger No. 1 (thickness = 700 μm) and finger No. 2 (thickness = 500 μm) was approximately the same for the both polymer materials ABS and PP. However, there are clearly differences in finger No. 3 (thickness = 300 μm) length especially in treatments 1, 5, 9 and 13 where holding pressure and injection velocity were at the minimum level: the finger length was very short compared to finger No. 1 and finger No. 2 for the same treatments, for both polymer materials ABS and PP. In addition, for ABS in treatment 2 and 10, where holding pressure and melt temperature were at the minimum level the finger length is shorter compared to finger No. 1 and finger No. 2 for the same treatments. For finger number 4 (thickness = 100 μm), the polymer flow was in fact obstructed so the finger length was very short for all 16 treatments, as shown in Figure 7 and Figure 8: the maximum flow length was approximately 5.5 mm (A/R = 55). Figure 7 and Figure 8 show also that PP yielded a flow length longer than with ABS; this was due to the different viscosities, particularly at higher processing temperatures. These observations are summarized in Figure 9, where Pareto charts show the significant factors for all the treatments, considering also the finger thickness and the materials. In particular, the finger thickness is the parameter with the highest effect for thin fingers (case (a) = 100 μm and 300 μm), whereas for thick fingers (case (b) = 500 μm and 700 μm), the thickness had lower effect than injection moulding process parameters such as holding pressure, injection speed and melt temperature. The fact that the parameter ‘Material’ had a low effect means that the two materials have a statistically similar behaviour as far as the flow length is concerned. This is particularly true for thicker fingers, where ‘Material’ was the parameter with the lowest effect (see Figure 9b).

**Figure 10:**
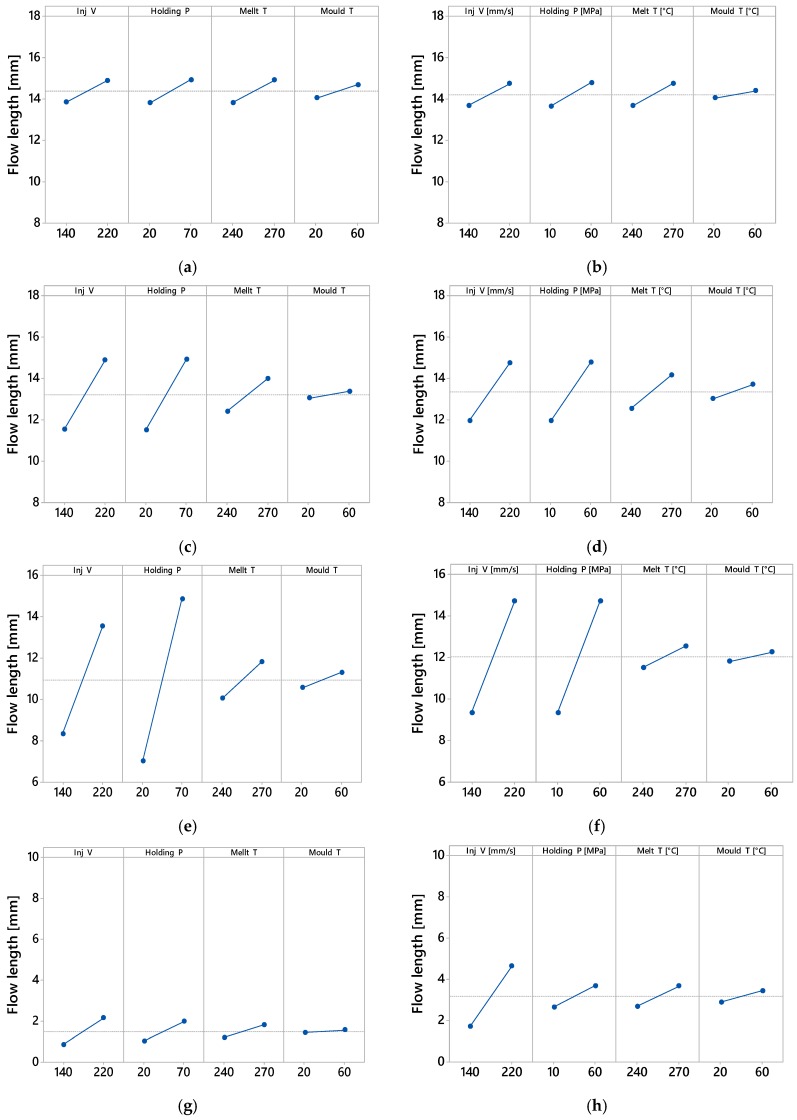
Main effect plot for the filling flow length of ABS and PP materials for the four different fingers. (**a**) Main effect plot ABS finger No. 1 (thickness = 700 μm); (**b**) Main effect plot PP finger No. 1 (thickness = 700 μm); (**c**) Main effect plot ABS finger No. 2 (thickness = 500 μm); (**d**) Main effect plot PP finger No. 2 (thickness = 500 μm); (**e**) Main effect plot ABS finger No. 3 (thickness = 300 μm); (**f**) Main effect plot PP finger No. 3 (thickness = 300 μm); (**g**) Main effect plot ABS finger No. 4 (thickness = 100 μm); (**h**) Main effect plot PP finger No. 4 (thickness = 100 μm).

When the main effects are considered, for finger No. 1 (thickness = 700 μm, A/R = 21) no parameters had any more influence on the finger length than another, for either material; finger length increased with the increase of all processing parameters, as shown on the main effect plot in Figure 10a,b. This was due to the fact that thickness of finger No. 1 is relatively high so the polymers could flow easily along its whole length.

With decreasing finger thickness, as in finger No. 2 (thickness = 500 μm, A/R = 30), the polymer flow became more difficult and a significant effect of process parameters appear. Holding pressure followed by injection velocity were the most influential parameters increasing finger length, for both materials, as shown in Figure 10c,d. The holding pressure applied at a switch over point before the no-flow temperature is reached allowed more material to fill the mould cavity before complete freezing of the melt. Furthermore, increasing velocity led to an increase in shear rate, which in turn decreased the viscosity of the polymer due to shear thinning, increasing the flow inside the mould cavity. With a further decrease in finger thickness, as in finger No. 3 (thickness = 300 μm, A/R = 50), the flow of polymer became more critical and therefore the optimal processing window was reduced. Specific processing parameters must be set, particularly with materials having higher viscosity, as for ABS in this case. For the ABS material, holding pressure, injection velocity and melt temperature were the most influential parameters increasing finger No. 3 length, as shown in Figure 10e. The effect of melt temperature is caused by the different viscosity characteristics of ABS at 270 °C (see Figure 1a): higher temperature decreased the viscosity and allowed the polymer to flow into the thinner features. For PP, which has a lower viscosity, holding pressure followed by injection velocity were the most influential parameters that increased finger length, as shown in Figure 10f. PP could flow in average 1.1 mm longer than ABS across the whole DOE, corresponding to a 10.0% longer flow length. For finger No. 4, (thickness = 100 μm thickness, A/R = 150), the flow length was short for both polymer materials and the maximum achieved aspect ratio was in average 15 for ABS and 32 for PP. The flow inside the very thin finger was very limited, especially for ABS, which has a higher viscosity. Holding pressure, injection velocity and melt temperature were the most influential parameters increasing finger No. 4 length for ABS and PP as shown in Figure 10g,h respectively. In average PP could flow 1.7 mm longer than ABS across the whole DOE, corresponding to a 112% longer flow length.

The experiments have demonstrated that for finger thicknesses of 500 μm and above both materials could reach the same flow length in average within the experimental process variability. For finger thicknesses of 300 μm and below, PP could flow longer than ABS with an increasing performance over ABS for increasingly thinner cavities.

For both materials, the relative importance of the process parameters was consistent in both amount of effect and trend across the whole DOE and for the four different thicknesses of the fingers. In particular it has been demonstrated that for decreasing thicknesses the effect of injection speed and packing pressure increased with respect to the effect of mould and melt temperature settings, that in fact remained relatively constant while the finger thicknesses decreased. This trend was verified for both PP and ABS, for all four processing parameters and four finger thicknesses. This means that in order to extend the filling flow length, the main process parameters of influence are injection speed and packing pressure, while increasing melt and mould temperature should be maintained within the recommended levels for the material as to avoid polymer degradation, long cycle time, deformation at ejection. This result is valid for different materials (in this case ABS and PP), as long as they are processed within the same process window and moulding the same geometry.

### 3.3. Factors Affecting Flash Formation

Flash is defined as additional unwanted material on the finished part, typically forming at the edge of injection moulded parts where melt flows from the cavity into thin gaps between parting surfaces of the injection moulding tool. Flash formation was investigated by Chen et al. in [28]. Flash was characterized in terms of length, closure time and pressure. However, the part considered in the experiments was a polycarbonate plate produced by conventional injection moulding having fairly large dimensions (length = 120 mm, width = 40 mm, thickness = 1.73 mm). Scientific research regarding flash formation is in fact still rather limited and in particular it has not been performed for miniaturized or micro moulded parts.

**Figure 11:**
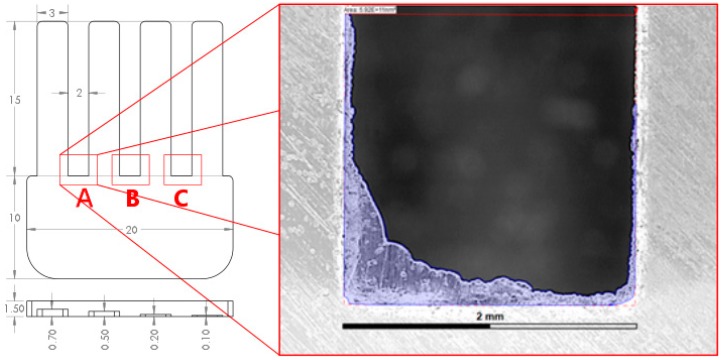
Flash formation inspection area with respect to sample design.

**Figure 12:**
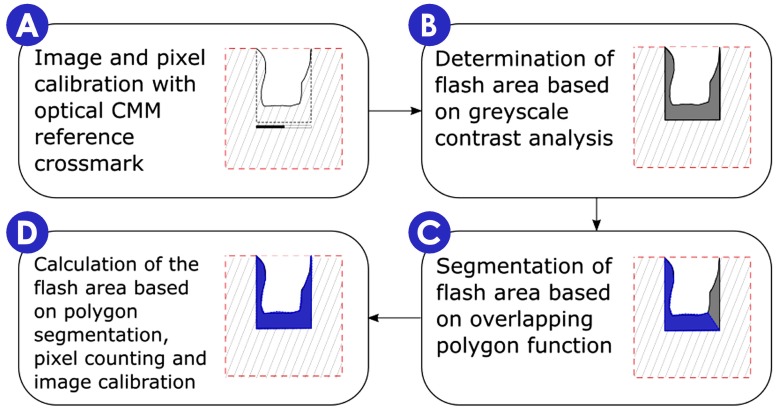
Workflow flash area measuring procedure.

In the present research, flash formation was investigated for both ABS and PP. In Figure 11 the squared regions on the sample design indicated the locations where flash occurred. Flash was measured on the portion designed with letter A, on all moulded parts produced in the DOE, to analyse the influence of moulding material and process parameters on the amount of flash produced during the trials. An optical quality control CNC CMM was used to capture calibrated microscopic photographs for evaluating the amount of flash formation in the same corresponding position of the moulded parts for each experimental run for both materials (see Figure 12). The optical CNC CMM was equipped with a 2× magnification lens and had a field of view of 3111 μm × 2327 μm in the X and Y directions respectively, with 768 × 576 pixels of 4 μm × 4 μm in size.

**Figure 13:**
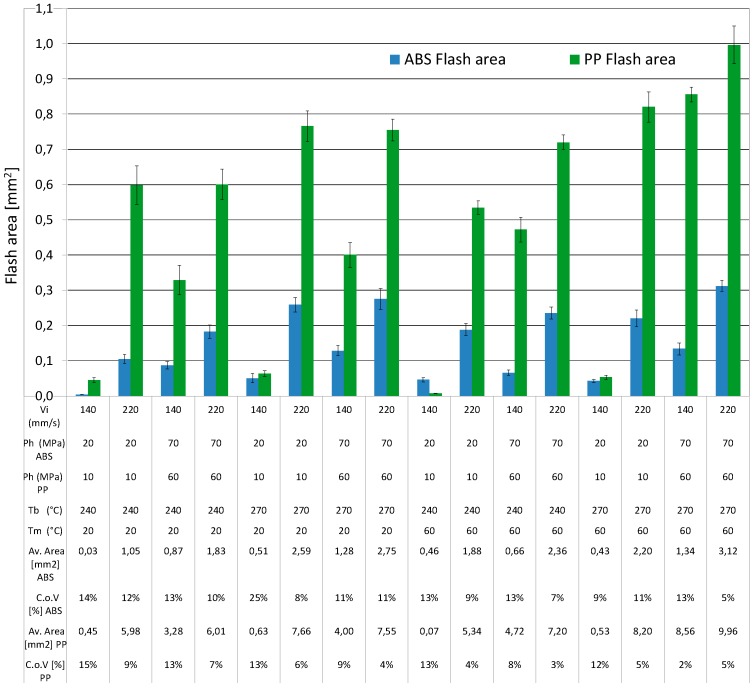
Average flash area for each experimental run for both materials ABS and PP. Note 1: error bars on histograms indicate experimental standard deviation (SD). Note 2: Coefficient of Variation COV% = average/SD × 100%.

The measurement uncertainty of the optical CMM when measuring areas was estimated to be 0.6%, meaning that for example when measuring an area of 1 mm^2^, the uncertainty is 0.06 mm^2^. This is considered to be sufficiently low with respect to the moulding process variability in terms of flash formation (see Figure 13) and its repeatability, which is at least one order magnitude higher. The area of the formed flash was measured by identifying on the calibrated images the contrast difference on the image due to the flash portion (steps A and B in Figure 12). With an image processing software (SPIP© by Image Metrology A/S, Hørsholm, Denmark), it was than possible to inscribe the flash section in a defined polygon (step C in Figure 12). At last the calculation of the area was possible as area of the defined polygon based on pixel counting and calibrated pixel area (step D in Figure 12). The robustness of the method was verified by replicating ten measurements of the same flash area. Afterwards, five independent measurements on five different parts for each of the 16 DOE process conditions were performed. An experimental relative standard deviation between 2% and 15% was observed in more than 95% of the cases (see Figure 13).

**Figure 14:**
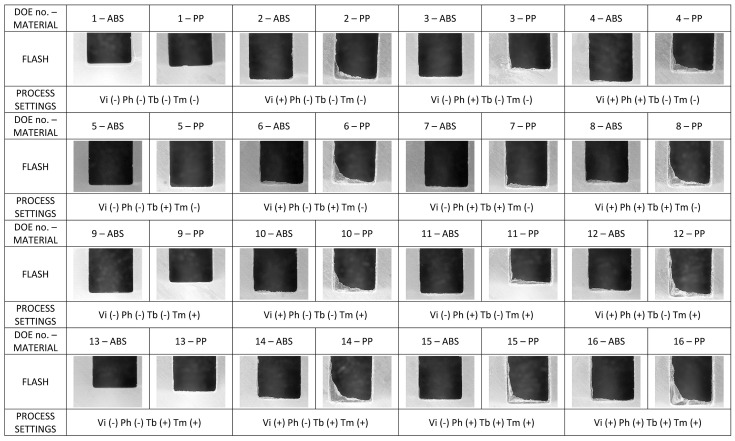
Images of PP (**right**) and ABS (**left**) flash formation in different process conditions at all the 16 DOE treatments. The gap between the left and right edges of the polymer features is 2.0 mm.

**Figure 15:**
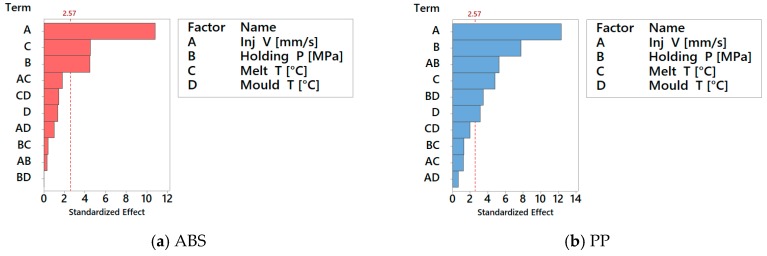
Pareto chart of the Standardized Effects on average Flash Area (α = 0.05) for (**a**) ABS and (**b**) PP material.

**Figure 16:**
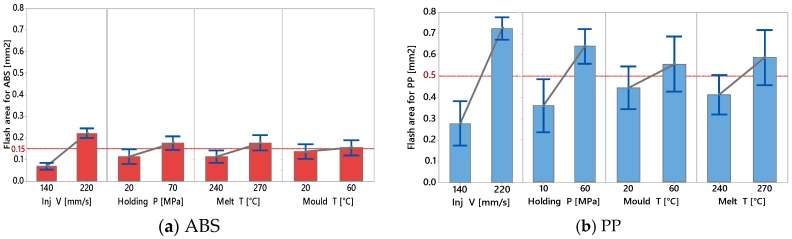
Main effect plot for Flash section area on (**a**) ABS and (**b**) PP.

Before assessing the DOE results, a preliminary optical imaging inspection showed that flash formation for the PP material was higher than for the ABS material for all 16 DOE treatments (see Figure 14). As shown in the Pareto charts in Figure 15a, the factors significance (consisting of a *p*-value lower than 5%) on the average flash formation for ABS was observed for injection velocity, holding pressure and melt temperature. These three process parameters were those with the highest main effect also for PP (see Figure 16b). Both factorial models were validated from a statistical point of view after a standardised residuals checking and the verification of an R-squared adjusted for the ABS model of 91.2% and 95.0% for the PP model was carried out.

All the main effects of the full factorial design are reported in Figure 16. The most important results can be seen from the difference in flash formation by changing the material from ABS to PP. Despite in Figure 10g,h an average higher finger replication was detected when using PP, Figure 16 shows that at the same time PP led to higher flash formation. In particular, the overall average flash area for PP was 3.3 times that the flash area for parts moulded with ABS (see Figure 16a,b). This variation can be explained by the different rheology of the two materials. As shown in Figure 1 ABS has a higher viscosity especially at high temperature setting which decreased the flowability (as shown in the flow length analysis) but in turn led to lower flash formation.

The same discussion is valid with respect to the high effect on the Injection Velocity. By directly affecting the shear rate and the viscosity, an injection velocity variation from 140 to 220 mm/s at least doubled the average flash area as shown by both Figure 16a,b and clearly indicated by the micrographs in Figure 13.

The effect of both the holding pressure and melt temperature was due once again in the reduction of the viscosity of the part and in applying higher pressure with the polymer in a particularly low viscosity state.

Despite the relatively higher effect of process parameters on flash formation by PP than by ABS, it is worth noticing that the relative importance among the parameters (namely the fact that the effect of injection speed is higher than that of packing pressure, which is higher than the effect of melt temperature and eventually of that of mould temperature) is consistent for both materials, for the considered geometry (see Figure 16a,b) and confirmed by the Pareto analysis (see Figure 15a,b).

Therefore, while to enhance high flow filling length high settings of injection speed and packing pressure are to be preferred, it must be considered in the moulding process design phase that those are the process parameters that at the same time also promote high flash formation.

## 4. Conclusions

Micro injection moulding is an important process in micro production engineering due to its low cost and high manufacturing volume. Manufacturing parts with high accuracy and quality is very important in polymer parts micro manufacture due to their high precision applications. The purpose of this study was to investigate the effect of several process parameters on multiple quality criteria for the resulting parts. In this work, the quality criteria considered were the part mass, the flow length and flash amount using micro fingers test structures.

Experimental results and statistical analysis showed that:

- Among the four process parameters considered in this research (injection velocity, holding pressure, melt temperature and mould temperature), results showed that the holding pressure and injection speed were the most effective on mass and flow length for both the used materials (ABS and PP) and the for all considered micro cantilever geometries.
- Flow length was inversely proportional to the thickness of the fingers high aspect ratio features. However, a non-linear behaviour was observed across the micro geometries analysed. For finger having thickness of 500 μm and above, both ABS and PP had similar flow length across the entire process window with similar filling behaviours. For finger thickness of 300 μm and below, PP had an increasingly better filling performance than ABS as the thickness decreased down to 100 μm. ABS flowability was severely challenged due to its higher viscosity with respect to PP. The decreasing of thickness gave raise to higher effect of injection speed and packing pressure in increasing the flow length, both for PP and ABS.
- The flash amount created when moulding the PP material was generally larger than the flash amount for the ABS material when considering corresponding processing conditions. Injection speed and holding pressure had higher effects than melt and mould temperature and revealed to be the most affective parameters on increasing the amount of flash for both material when set at high levels. The area of flash generated when moulding PP was in average 3.3 larger than that of ABS. However, the relative importance of the four process parameters in promoting flash formation was the same for ABS and PP, indicating similarity among the two materials in their relative sensitivity to changes in moulding parameters settings.

In conclusion, in order to replicate high quality parts with sub-mm thickness and high aspect ratio features, correct adjustment of the process parameters is crucial. In particular, holding pressure and injection velocity proved to be very important for part mass and flow length accuracy. Conversely, injection velocity and holding pressure had an undesirable effect on the amount of flash formation. Indeed, the choice of the polymer material had a large effect on the quality of the part in terms of flow length and flash formation, particularly for the smallest thicknesses (300 μm and 100 μm in this research). In spite of the fact that a less viscous polymer such as PP can replicate features with higher aspect ratio, it also promotes higher flash formation. For this reason, especially when the geometrical aspect ratio increases, it is crucial to identify the optimum set of moulding parameters to produce a high-quality part in relation to the corresponding selected polymer.

## Figures and Tables

**Table 1:** Two-level four-factor full-factorial design.

Standard Order	V_i_	P_h (PP/ABS)_	T_b_	T_m_
1	−(140 mm/s)	−(10/20 MPa)	−(240 °C)	−(240 °C)
2	+(220 mm/s)	−(10/20 MPa)	−(240 °C)	−(240 °C)
3	−(140 mm/s)	+(60/70 MPa)	−(240 °C)	−(240 °C)
4	+(220 mm/s)	+(60/70 MPa)	−(240 °C)	−(240 °C)
5	−(140 mm/s)	−(10/20 MPa)	+(270 °C)	−(240 °C)
6	+(220 mm/s)	−(10/20 MPa)	+(270 °C)	−(240 °C)
7	−(140 mm/s)	+(60/70 MPa)	+(270 °C)	−(240 °C)
8	+(220 mm/s)	+(60/70 MPa)	+(270 °C)	−(240 °C)
9	−(140 mm/s)	−(10/20 MPa)	−(240 °C)	+(270 °C)
10	+(220 mm/s)	−(10/20 MPa)	−(240 °C)	+(270 °C)
11	−(140 mm/s)	+(60/70 MPa)	−(240 °C)	+(270 °C)
12	+(220 mm/s)	+(60/70 MPa)	- (240 °C)	+(270 °C)
13	−(140 mm/s)	−(10/20 MPa)	+(270 °C)	+(270 °C)
14	+(220 mm/s)	−(10/20 MPa)	+(270 °C)	+(270 °C)
15	−(140 mm/s)	+(60/70 MPa)	+(270 °C)	+(270 °C)
16	+(220 mm/s)	+(60/70 MPa)	+(270 °C)	+(270 °C)
